# N-acylated Peptides Derived from Human Lactoferricin Perturb Organization of Cardiolipin and Phosphatidylethanolamine in Cell Membranes and Induce Defects in *Escherichia coli* Cell Division

**DOI:** 10.1371/journal.pone.0090228

**Published:** 2014-03-03

**Authors:** Dagmar Zweytick, Bostjan Japelj, Eugenia Mileykovskaya, Mateja Zorko, William Dowhan, Sylvie E. Blondelle, Sabrina Riedl, Roman Jerala, Karl Lohner

**Affiliations:** 1 Institute of Molecular Biosciences, Biophysics Division, University of Graz, Graz, Austria; 2 Department of Biotechnology, National Institute of Chemistry, Ljubljana, Slovenia; 3 Department of Biochemistry and Molecular Biology, University of Texas Medical School-Houston, Houston, Texas, United States of America; 4 Department of Biochemistry, Torrey Pines Institute for Molecular Studies, San Diego, California, United States of America; 5 Centre of Excellence EN-FIST, Ljubljana, Slovenia; National Research Council of Italy, Italy

## Abstract

Two types of recently described antibacterial peptides derived from human lactoferricin, either nonacylated or N-acylated, were studied for their different interaction with membranes of *Escherichia coli in vivo* and in model systems. Electron microscopy revealed striking effects on the bacterial membrane as both peptide types induced formation of large membrane blebs. Electron and fluorescence microscopy, however demonstrated that only the N-acylated peptides partially induced the generation of oversized cells, which might reflect defects in cell-division. Further a different distribution of cardiolipin domains on the *E. coli* membrane was shown only in the presence of the N-acylated peptides. The lipid was distributed over the whole bacterial cell surface, whereas cardiolipin in untreated and nonacylated peptide-treated cells was mainly located at the septum and poles. Studies with bacterial membrane mimics, such as cardiolipin or phosphatidylethanolamine revealed that both types of peptides interacted with the negatively charged lipid cardiolipin. The nonacylated peptides however induced segregation of cardiolipin into peptide-enriched and peptide-poor lipid domains, while the N-acylated peptides promoted formation of many small heterogeneous domains. Only N-acylated peptides caused additional severe effects on the main phase transition of liposomes composed of pure phosphatidylethanolamine, while both peptide types inhibited the lamellar to hexagonal phase transition. Lipid mixtures of phosphatidylethanolamine and cardiolipin revealed anionic clustering by all peptide types. However additional strong perturbation of the neutral lipids was only seen with the N-acylated peptides. Nuclear magnetic resonance demonstrated different conformational arrangement of the N-acylated peptide in anionic and zwitterionic micelles revealing possible mechanistic differences in their action on different membrane lipids. We hypothesized that both peptides kill bacteria by interacting with bacterial membrane lipids but only N-acylated peptides interact with both charged cardiolipin and zwitterionic phosphatidylethanolamine resulting in remodeling of the natural phospholipid domains in the *E. coli* membrane that leads to defects in cell division.

## Introduction

Since antibiotic resistance is still an increasing health problem, investigation of new antibiotics such as cationic antimicrobial peptides (AMPs) is of great interest. Different than non-peptide-based antibiotics, which usually inhibit cell wall and protein biosynthesis or DNA replication, AMPs act predominantly without binding to specific receptors but interact directly with the lipid matrix of bacterial cell membranes [*1*]. Cationic peptides target mainly negatively charged lipids exposed on the surface of bacterial membranes. We recently reported the different effects of lactoferricin-derived peptides, nonacylated and N-acylated, on *E. coli* membranes and on model liposomes composed of bacterial lipids [*2*]. Perturbation of the lipid organization in liposomes composed of negatively charged lipids such as phosphatidylglycerol or total *E. coli* lipid and binding to lipopolysaccharides (LPS) were shown to be enhanced by hydrophobic modifications of the peptide by N-acylation. However, zwitterionic lipids such as phosphatidylcholine or phosphatidylethanolamine (PE) were also partially affected by such peptide derivatives [*2*]; [*3*]. The cell envelope of Gram-negative bacteria, like *E. coli*, consists of a cytoplasmic or inner membrane (IM), a peptidoglycan layer, and an outer membrane (OM) [*4*]. The OM itself harbors proteins, phospholipids and LPS [*4*]. A number of AMPs (e.g. tachyplesin, magainin and cecropin A) first interact with the negatively charged LPS to form a complex [*5*], then transfer across the OM to the periplasmic space [*6*], and finally perform their lethal effect by interaction with the IM, which is mainly composed of PE, PG and cardiolipin (CL) [*7*]. Numerous studies demonstrated that AMPs interfere with the integrity of bacterial membranes via diverse mechanisms (for reviews see [*5*]; [8]; [*9*]). The most frequently discussed modes of action include the formation of toroidal pores [*10*]; [*11*] and the coverage of the membrane surface by peptides (carpet model [*12*]). The capability of AMPs to cluster anionic lipids is also described to be a mechanism applied by cationic peptides [*13*]–[*15*]. However the mechanism of the final killing step appears to depend on peptide concentration and type [*16*] as well as on their structure in presence of membrane [*17*]–[*20*]. For instance, it was shown that the structure of the antimicrobial center of bovine lactoferricin (LFcinB - RRWQWR-NH2) bound to sodium dodecyl sulfate (SDS) micelles is amphipathic with the Trp side chains separated from the Arg residues [*19*]. At concentrations below the minimum inhibitory concentration (MIC) membrane blebbing and detachment of IM and OM were observed in the presence of peptide LL-37 and cecropin B, while membrane lysis of *E. coli* occurred at their MIC [*16*]. In the case of human lactoferricin (hLFcin) derivatives, we previously reported detachment of OM and IM and protrusions at MIC, and established that this severe membrane effect led to bacterial killing before visible membrane lysis [*2*]. Fragments of LFcinB were shown to localize in the cytosol of *E. coli* [*21*], where they affected protein and DNA synthesis [*22*]. After one hour of exposure to LFcinB at MIC, a profound effect on cell morphology of *E. coli* was observed. The LFcinB exposed cells became filamentous and elongated and did not appear to be dividing in a regular manner [*22*]. Similar effects were observed when *E. coli* was incubated with the antibiotic bicyclomycin, which inhibits septum formation and converts the cells to filamentous forms. The antibiotic also induced high undulation and numerous blebs of the outer membrane [*23*]. Such formation of filamentous *E. coli* was also observed in the present study though only in the presence of N-acylated peptides.

Besides proteins that have been reported to be required in the process of cell division and septum formation in prokaryotes [*24*]; [*25*], the specific involvement of phospholipids in this membrane-associated process has also been investigated [*26*]–[*29*]. For example, PE was proposed to play an important direct or indirect role at some stage of the cell division cycle, since the *E. coli* mutant *pss-93*, which lacks PE grows as filamentous cells and is apparently defective in cell division [*27*]. In this mutant FtsZ rings localized properly at division sites but failed to constrict [*27*]. PE is a lipid able to undergo a bilayer-to-non-bilayer transition, a property that might be essential in cell division processes [*30*]; [*31*]. Similarly CL is reported to play a role in cell division, e.g. via formation of membrane domains that seem to participate in this process [*28*]. However, though CL is also able to undergo a bilayer-to-non-bilayer transition in the presence of divalent cations, it was shown not to completely compensate for PE in the PE lacking mutant *pss-93* [*32*]. To gain further insight into the multiple mechanisms of action of antimicrobial peptides, the impact of nonacylated and N-acylated hLFcin derivatives on morphology and cell division of *E. coli* was investigated and correlated with their effects on the phospholipids PE and CL. The results indicate that N-acylated peptides act via interaction with these lipids by a different mechanism than their nonacylated parent peptide. The former elicits a stronger perturbation of membrane lipid organization and also inhibits the cell division process.

## Experimental Procedures

### Lipids and peptides

1-palmitoyl-2-oleoyl-sn-glycero-3-phosphoethanolamine (POPE) and tetramyristoylcardiolipin (TMCL) were purchased from Avanti Polar Lipids, Inc. (USA), and used without further purification.

The amidated peptides LF11-322 (PFWRIRIRR-NH_2_, M = 1298.6 g/mole), its N-6-methyloctanoyl derivative 6-MO-LF11-322 (CH_3_CH_2_-CH_2_(CH_3_)-(CH_2_)_4_-CO-NH-PFWRIRIRR-NH_2_, M = 1438.9 g/mole) and LF11-215 (FWRIRIRR-NH_2_, M = 1201,5 g/mole) and its N-octanoyl derivative O-LF11-215 (CH_3_-(CH_2_)_6_-CO-NH-FWRIRIRR-NH_2_, M = 1327,7 g/mole) were purchased from PolyPeptide Laboratories (San Diego, CA, USA) (see Table 1).Peptides were dissolved in phosphate buffered saline (PBS, 20 mM NaPi, 130 mM NaCl, pH 7.4), if not otherwise indicated at a concentration of 3 mg/ml before each experiment.

**Table 1:** Primary structure, hydrophobicity and biological activity of LF11 [*2*]; [*3*] derived peptides and N-acylations thereof.

peptide designation	N-acyl group	amino acid sequence	Net charge	MIC*_E. coli_* a [µg/ml]
LF11-215		F W R I R I R R-NH_2_	+5	16*–*32
O-LF11-215	octanoyl	F W R I R I R R-NH_2_	+4	10
LF11-322		P F W R I R I R R-NH_2_	+5	8*–*16
6-MO-LF11-322	6-methyl-octanoyl	P F W R I R I R R-NH_2_	+4	8*–*16

a Minimal inhibitory concentration (MIC) against *E. coli* ATCC 25922 were determined as peptide concentration resulting in less than 2% growth following an overnight incubation in Mueller Hinton medium at 37°C in the presence of 5×10^5^ CFU/ml.

### Assays for antimicrobial activity

Peptides antimicrobial activity against *E. coli* ATCC 25922 (5*10^5^ CFU/ml) was tested using susceptibility micro dilution assays according to NCCLS (National Committee for Clinical Laboratory Standards) approved guidelines and was determined as described elsewhere [*33*]. All assays were performed in duplicate and three times.

### Electron microscopy

*E. coli* O:111 was grown overnight at 37°C, transferred to fresh medium and grown to exponential phase, and further diluted with fresh LB (lysogeny broth) medium to a final density of 4×10^7^ CFU/ml (colony-forming unit/ml). The cells were exposed to peptides at a concentration corresponding to the MIC for 1 h at room temperature. The cells were harvested by centrifugation and fixed by immersion in 4% glutaraldehyde in 0.1 M sodium phosphate buffer. The bacterial cultures were incubated 1 h on ice and then dehydrated in an ethanol gradient and stored in 100% ethanol. Specimens in100% ethanol were critical point dried in a CO_2_ atmosphere and mounted on aluminum stubs and finally coated with gold particles in a puttering process. The specimens were examined by a Field-Emission Scanning Electron Microscope - Supra 35 VP Carl Zeiss.

### Fluorescence microcopy; CL staining

*E. coli* strain W3110 (http://ecoliwiki.net/colipedia/index.php/Strain:W3110) was used. Overnight culture grown at 37°C in LB was inoculated into fresh LB medium and grown to an OD_600_ of 0.5. In one set of experiments, 10-N-nonyl-3,6-bis (dimethylamino) acridinium bromide (10-N-nonyl acridine orange, NAO) was added to a final concentration of 500 nM. When the cells reached OD_600_ of 1.5 they were diluted with fresh LB medium to OD_600_ 0.15 and incubated with shaking for 1 h at room temperature with 2xMIC of peptides LF11-215 or O-LF11-215 (dissolved in 0.1% acetic acid), or 0.1% of acetic acid used as control. The cells were then harvested by centrifugation, re-suspended in a small volume of fresh LB media and viewed using a fluorescence microscope (see below). In another set of experiments, staining was performed after incubation with peptides: Overnight culture was grown at 37°C in LB and inoculated into fresh LB medium. When the cells reached an OD_600_ of 1.0 they were diluted with fresh LB medium to OD_600_ of 0.2 and incubated for 1 h at 30°C with 2xMIC of peptides O-LF11-215 or LF11-215, or 0.1% acetic acid as control. Then 500 nM NAO (final concentration) was added to each sample, and cells were incubated for another 1 h at 30°C. Cells from both samples were immobilized on microscope slide cover glasses with poly-L-lysine and viewed using an Olympus BX60 microscope with an 100× oil-immersion objective and FITC (fluorescein isothiocyanate) filter. Images were captured with a light-sensitive Photometrics Cool-Snap FX cooled charge-coupled device camera driven by QED image capturing software and saved as Adobe Photoshop TIF files.

### Preparation of liposomes for differential scanning calorimetry (DSC)

Stock solutions of POPE were prepared in CHCl_3_/CH_3_OH (9:1, v/v) and of TMCL in CHCl_3_ and stored at −18°C if necessary. For preparation of lipid films of mixtures of POPE/TMCL (80∶20, w/w) the respective amount of the lipid stock solutions were combined. Lipid films of 1 mg of the respective lipid (or mixture thereof) dissolved in organic solvent were dried under nitrogen and evaporated over night to remove residual traces of organic solvent.

Aqueous dispersions of lipids of 0.1 wt.% in PBS-buffer (20 mM NaPi, 130 mM NaCl, pH 7.4) were prepared before measurement in the presence (lipid-to-peptide molar ratio of 25∶1) and absence of peptides as described elsewhere [*34*]; [*35*]. Briefly, hydration temperature for preparation of POPE liposomes was 30°C and for TMCL liposomes 50°C, samples were vortexed every 15 minutes during 2 hours of preparation time. The hydration of the lipid mixture POPE/TMCL 80∶20 was performed at 45°C for 2 hours by vortexing accompanied by one freeze-thaw cycle every 15 minutes.

### Differential scanning calorimetry (DSC)

DSC experiments were performed with a differential scanning calorimeter (VP-DSC), from MicroCal, Inc. (Northampton, MA, USA). Samples were degassed before measuring. Heating scans were performed at a scan rate of 30°C/h with a final temperature approximately 10°C above the main transition temperature (T_m_) or hexagonal phase transition temperature (T_HII_) and cooling scans at the same scan rate with a final temperature about 20°C below T_m_. The heating/cooling cycle was repeated twice, pre-scan thermostating was allowed for 30 minutes for the heating scans and 1 minute for the cooling scans. Enthalpies were calculated by integrating the peak areas after normalization to phospholipid concentration and baseline adjustment using the MicroCal Origin software (VP-DSC version).

### Nuclear Magnetic Resonance (NMR) Spectroscopy

2.3 mM O-LF11-215 was prepared in 222.6 mM d25-SDS (sodium dodecyl-d25 sulfate), 20 mM sodium phosphate pH = 5.1, 8% D_2_O. 2.4 mM O-LF11-215 was prepared in 200.4 mM d38-DPC (dodecylphosphocholine-d38), 20 mM sodium phosphate pH = 5.4, 8% D_2_O. In all samples, an excess of micelle to peptide concentration was present. The spectra were recorded at 30°C on a Varian Unity INOVA 600 MHz spectrometer equipped with a 5 mm ^1^H[^13^C/^15^N] Pulse Field Z-Gradient Triple Probe. 2-D homonuclear TOCSY (total correlation spectroscopy) and NOESY (nuclear Overhauser enhancement spectroscopy) spectra were acquired with 2048×512 complex data points in both dimensions for SDS (sodium dodecylsulfate) and DPC (dodecylphosphocholine) spectra, respectively. TOCSY spectra of O-LF11-215 samples were recorded at mixing time of 80 ms, while NOESY spectra were recorded at mixing times of 100 and 150 ms. Water suppression was achieved using WET or WATERGATE solvent suppression schemes [*36*]; [*37*]. NMR data of the O-LF11-215 spectra were processed using Sparky 3.110 (http://www.cgl.ucsf.edu/home/sparky/). Resolution enhancement was achieved by apodization of the free induction decay with a shifted square sine-bell window function. Chemical shifts were referenced to the internal standard DSS (4,4-dimethyl-4- silapentane-1-sulfonate). Calibration of volumes and conversion of volumes into DYANA (DYnamics Algorithm for NMR Applications) restraints for upper bounds was achieved using the nmr2st program [*38*]. Two well resolved germinal methylene proton cross-peaks of Trp2:HH2-HZ2 and Trp2:HE3-HZ3 signals were used for distance calibration of O-LF11-215 sample spectra. The experimental distance constraints were then employed to generate peptide conformers using 6000 steps of simulated annealing in torsion angle space implemented in the program DYANA version 1.5 [*39*]. Since the octanoyl residue on the N-terminus was not recognized by DYANA software the Phe-C8 residue was created using nmr2st software. Out of 200 calculated structures 10*–*20 structures with the lowest target function were kept for further minimization. DISCOVER (Accelrys) using the cvff force field was used to energy-minimize the structures using the linear distance-dependent dielectric model. None of the residues were in the disallowed region of the Ramachandran plot. The quality of the structures was checked with PROCHECK-NMR [*40*]. Structures were visualized and analyzed with MOLMOL [*41*].

5- and 16-doxyl stearate molecules were used as paramagnetic probes to measure the depth of insertion of O-LF11-215 into the SDS and DPC micelles, respectively. A pair of reference and DSA (doxylstearic acid) spectra was recorded for 5- and 16-DSA (16-doxylstearic acid) of the O-LF11-215 peptide in SDS and DPC micelles. The O-LF11-215 SDS (5-DSA, 5-doxylstearic acid) reference sample contained 1.16 mM peptide, 111.3 mM d25-SDS, 10 mM sodium phosphate, pH = 5.5 and 8% D_2_O. 0.35 mg 5-DSA was dissolved to give the O-LF11-215 SDS 5-DSA sample. O-LF11-215 SDS (16-DSA) reference sample contained 1.56 mM peptide, 169.0 mM d25-SDS, 10 mM sodium phosphate, pH 5.5 and 8% D_2_O. 0.35 mg 16-DSA was dissolved to give the O-LF11-215 SDS 16-DSA sample. The O-LF11-215 DPC 5-DSA reference sample contained 1.62 mM peptide, 111.6 mM d38-DPC, 20 mM sodium phosphate, pH = 5.7 and 8% D_2_O. The O-LF11-215 DPC 16-DSA reference spectrum contained 1.56 mM peptide, 169.0 mM d38-DPC, 20 mM sodium phosphate, pH = 5.8 and 8% D_2_O. 0.35 mg 5- or 16-DSA was dissolved in the reference sample for the O-LF11-215 DPC 5- or 16-DSA samples respectively.

The experiments were performed by first recording the reference NOESY spectra of O-LF11-215 in complex with SDS and DPC micelles. 5- and 16-DSA were added to the solution causing paramagnetic relaxation enhancement of the nuclei in close vicinity. 5-DSA has paramagnetic center close to the surface, whereas 16-DSA has paramagnetic center buried into micelle interior- in this way selective NMR signal broadening can be achieved. Paramagnetic relaxation is reversely proportional to the sixth power of the distance between nucleus and paramagnetic center [*42*]. NOESY spectra of the reference and DSA samples in d25-SDS and d38-DPC micelles were acquired at 150 ms time using 2048×512 complex data points at 30°C. H^N^-H^α^ intensities were measured in spectra followed by normalization of H^N^-H^α^intensities with addition of DSA by the H^N^-H^α^ intensities in the reference spectra without addition of DSA.

## Results

In the present study we investigated the different mechanisms by which nonacylated and N-acylated lactoferricin derived peptides (Table 1) interact with bacterial membranes in *in vivo* and in model systems to gain insight into peptide effects on lipids that impair essential steps of bacterial cell proliferation.

### Impact of peptides on morphology of E. coli membranes

**Figure 1:**
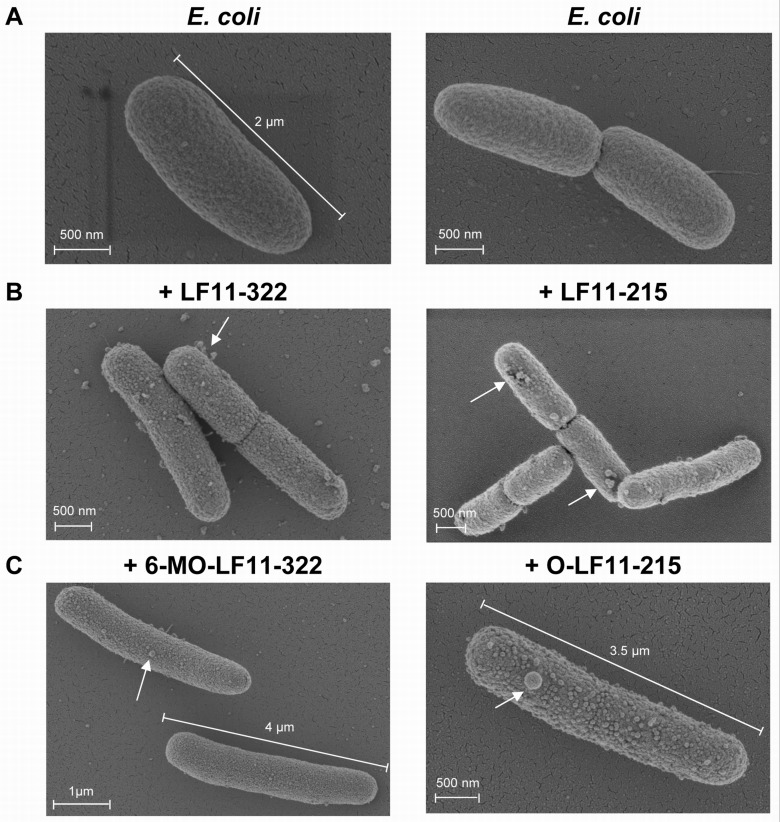
Peptide effect on bacterial membrane morphology. Scanning electron microscopy pictures taken of *E.coli* cells in the absence (A) and in the presence of nonacylated peptides LF11-322 (B left) and LF11-215 (B right), and N-acylated peptides 6-MO-LF11-322 (C left) and O-LF11-215 (C right) at the MIC level. Cells were incubated for 1 hour. Arrows indicate bleb formation (B and C) Upon incubation with N-acylated peptides partially elongated *E. coli* cells appeared (C).

*E. coli* cells were treated for one hour with peptides derived from lactoferricin (Table 1) at their respective MIC and then visualized by scanning electron microscopy. Bacterial membrane lysis was not detected under the experimental condition in agreement with our earlier observations [*2*]. This may in part be attributed to the necessity to work with a cell density of *E. coli* 100-fold higher than the one used in the assessment of antimicrobial activity and therefore resulting in lower effective concentration of peptides per bacterial cell. Nevertheless, all peptides exhibited profound effects on the morphology of *E. coli* (Figure 1B and 1C). Formation of numerous blebs was observed in the presence of all peptides (Figure 1 indicated by arrows) and was most obvious in case of LF11-215 and its N-acyl derivative O-LF11-215. In addition, in the presence of the N-acylated peptides 6-MO-LF11-322 and O-LF11-215 partial formation of larger cells with lengths between 3-4 µm was observed (Figure 1C, additional bars) as compared to ∼2 µm for untreated control cells (Figure 1A) suggesting inhibition of cell division.

### Impact of peptides on lipid domains in E. coli membranes

**Figure 2:**
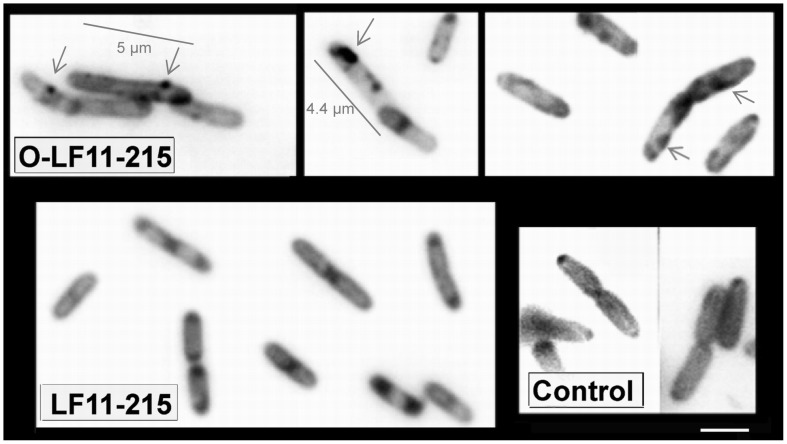
Cardiolipin domains – NAO staining before peptide treatment. Fluorescence microscopy of *E. coli* strain W3110 stained with 10-N-nonyl acridine orange (NAO) demonstrating localization of CL domains. NAO staining was performed before treatment with peptides. After staining cells were incubated with peptides LF11-215 or O-LF11-215 or 0.1% acetic acid (control). For details, see “Experimental Procedures”. Cells were immobilized on a microscope slide cover glass with poly-L-lysine and viewed using Olympus BX60 microscope with a 100× oil-immersion objective and FITC filter. Bar, 2 µm. Images were processed in Photoshop and converted into a *grayscale* with inversion resulting in the *black contour - white background images*. Arrows (upper pictures) show non-typical CL domains appearing distant from septum and poles in elongated with sizes mainly larger than 4 µm (respective bars) N-acylated peptide O-LF11-215 treated cells. Sizes of nonacylated peptide LF11-215 treated *E. coli* cells seemed in the range of 2 µm; CL domains in these cells are located at septum and poles and unmodified compared to wild type. For details, see “Experimental Procedures”. Cells were immobilized on a microscope slide cover glass with poly-L-lysine and viewed using Olympus BX60 microscope with a 100× oil-immersion objective and FITC filter. White bar, 2 µm. Arrows indicate altered CL domain formation in the treated by N-acylated peptide O-LF11-215 mainly elongated or filamentous cells.

**Figure 3:**
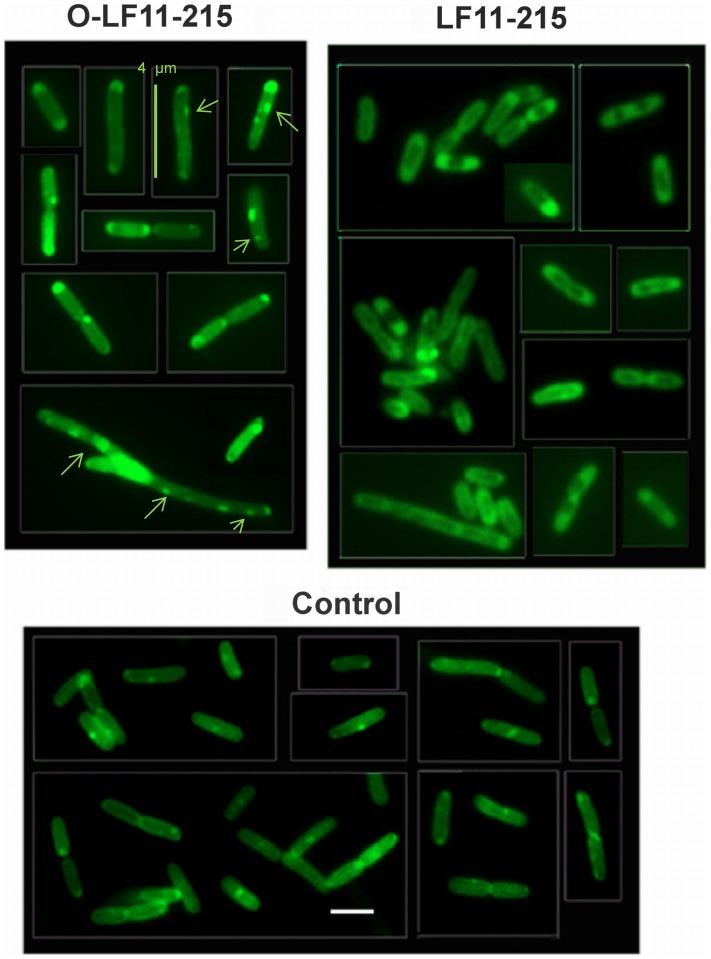
Cardiolipin domains – NAO staining after peptide treatment. Fluorescence microscopy of *E. coli* strain W3110 stained with 10-N-nonyl acridine orange (NAO) demonstrating localization of CL domains. NAO staining was performed after incubation of the cells with peptides O-LF11-215 and LF11-215, and controls consisting of aliquots of 0.1% acetic acid. For details, see “Experimental Procedures”. Cells were immobilized on a microscope slide cover glass with poly-L-lysine and viewed using Olympus BX60 microscope with a 100× oil-immersion objective and FITC filter. White bar, 2 µm. Arrows indicate altered CL domain formation in the treated by N-acylated peptide O-LF11-215 mainly elongated or filamentous cells.

Recently CL has been shown to play a role in cell division, e.g. via formation of membrane domains that seem to participate in this process [*28*]. Therefore, the dye NAO was used to detect possible effects of the peptides on the distribution of CL within the cell membrane. NAO is known to specifically bind to anionic phospholipids by an interaction between its quaternary amine and the phosphate residue of the phospholipids and by an intercalation of its hydrophobic acridine moiety into the membrane bilayer [*43*]. Because CL comprises two phosphate groups the dye forms a dimer yielding a much higher affinity of NAO for CL than with other (monoacidic) phospholipids [*43*]. Since peptide interaction with CL itself could change NAO staining by a competitive reaction, two types of experiments were designed, one adding the peptide before staining (Figure 2) and one adding it afterwards (Figure 3). When NAO staining was performed with “normal” *E. coli* exhibiting wild-type phospholipid composition Mileykovskaya et al. [*26*] observed that CL mainly localized to poles and septum areas. Cells were of normal size and cell division was not influenced by NAO staining. This could also be observed in the control experiments shown in Figure 2 and 3 using wild-type *E. coli* stained in the absence of peptides.

Similarly to what was observed in the electron microscopy (Figure 1), fluorescence microscopy of *E. coli* (Figure 2) following incubation with N-acylated peptides revealed oversized cells supporting the idea that such peptides exhibit an inhibiting effect on cell division, which seems at least partially to be driven by their effect on CL domains. Furthermore, NAO-staining before peptide incubation clearly showed that O-LF11-215 significantly affects the distribution of CL domains, which after this treatment located not only at the septum and poles but exhibited a more overall distribution along the cell membrane (Figure 2, arrows). Besides, staining seemed to be more intense. On the contrary, LF11-215 treated cells resembled mainly the control cells with however again more intense stained domains, but the main distribution of CL remained at septum and poles.

Figure 3 shows cells upon NAO-staining after peptide incubation, which is expected to allow the peptide to act more efficiently on the membrane. Both peptides, non- and N-acylated, indeed induced a brighter staining relative to the untreated control. Since the cells were treated with peptides before staining, membrane permeabilization by the peptides could cause a better uptake of NAO. Similarly to the first set of experiments, O-LF11-215 treatment caused higher than average sized cells (Figure 3 additional bars) and CL domains were distributed over the whole cell surface (Figure 3, arrows). LF11-215 treated cells again showed wild-type distribution of CL domains. In both types of experiments, the addition of N-acylated peptides suggested a direct influence of these peptides on CL domain formation and localization.

### Effects of peptides on the thermotropic phase behavior of bacterial lipids TMCL and POPE

**Figure 4:**
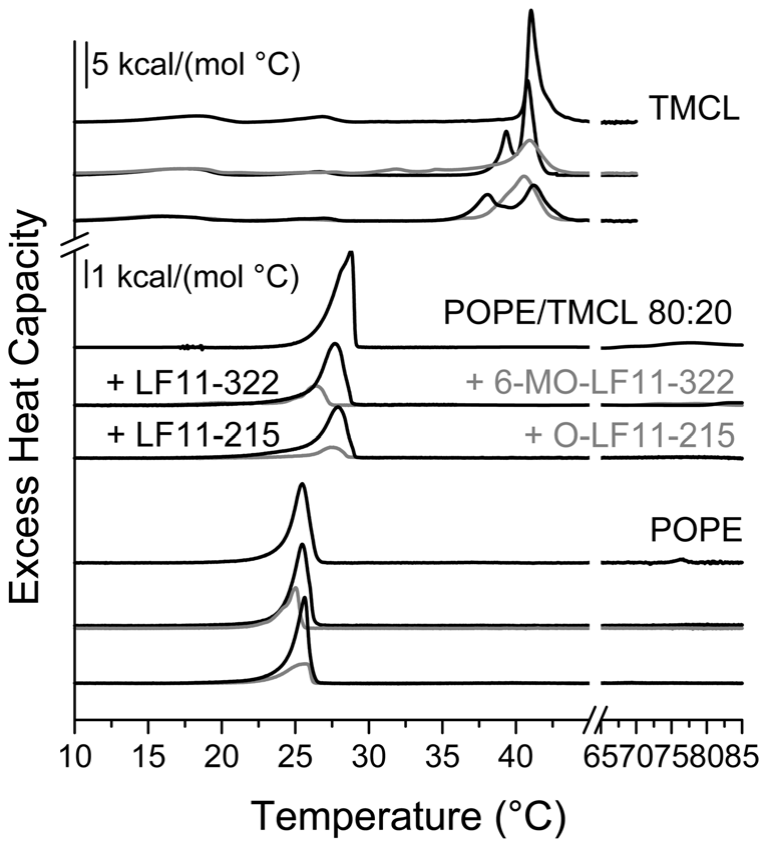
Model studies with TMCL and POPE. DSC thermograms of TMCL, POPE/TMCL 80:20 (w/w) and POPE in the absence and presence of peptides (lipid-to-peptide molar ratio of 25:1). For clarity, the DSC curves were displayed on the ordinate by arbitrary units. Scan rate was 30°C/h. N-acylated peptides are shown in gray. For analyzed data see Table 2.

Since CL appears to be involved in cell division, e.g. via formation of membrane domains [*28*] it is an interesting model to study the mechanistic differences observed for the two peptide types. Accordingly the effects of the four peptides on the thermotropic phase behavior of aqueous dispersions of the quadruple-chained, anionic phospholipid tetramyristoylcardiolipin (TMCL) were studied. Results are shown in Figure 4 and summarized in Table 2. In agreement with earlier calorimetric studies [*35*]; [*44*] three thermotropic phase transitions were observed upon heating. The two lower temperature transitions are of low enthalpy and cooperativity. They were assigned previously to phase transitions between two subgel domains (L_c’_ and L_R1_) and a subgel domain coexisting with a lamellar tilted gel phase (L_c’_ and L_β_) at 18.3°C, which transform into a tilted lamellar gel phase (L_β’_) at 26.8°C [*44*]. At 41°C a highly cooperative transition to a lamellar fluid phase occurred. Only negligible effects were observed on the lowest temperature transition upon addition of the four peptides at a lipid-to-peptide molar ratio of 25∶1. The second low temperature transition, on the other hand, was more affected, being more pronounced for the N-acylated peptides, as indicated by a strong reduction of the respective enthalpy (ΔH_pre2_) by more than 50%. Furthermore, nonacylated and N-acylated peptides had different effects on the main transition. A severe loss of cooperativity, as indicated by a strong broadening of the main transition, was observed upon incubation with the N-acylated peptides, while incubation with the nonacylated peptides LF11-322 and LF11-215 caused splitting of the main transition in an apparently peptide-enriched TMCL domain melting at lower temperatures (39.3°C for LF11-322 and 38.1°C for LF11-215) and a remaining mainly peptide unaffected TMCL domain melting near the transition temperature of pure TMCL.

**Table 2:** Thermodynamic parameters of TMCL, POPE/TMCL 80:20 and POPE in the absence and presence of LF11-derived peptides at a lipid-to-peptide molar ratio of 25:1.

	ΔH_pre1/2_ [kcal/mol]	T_pre1/2_ [°C]	ΔH_m_ [kcal/mol]	T_m_ [°C]	ΔT_1/2_ [°C]
TMCL	3.8/2.0	18.3/26.8	14.8	41.04	0.80
LF11-322	4.2/1.3	17.5/26.6	4.9/10.3	39.3/40.8	0.88/0.84
6-MO-LF11-322	3.2/0.5	17.9/27.5	12.4	40.9	2.01
LF11-215	3.1/1.0	16.3/27.0	6.1/8.2	38.1/41.2	2.04/2.04
O-LF11-215	4.1/0.9	15.7/25.6	14.1	40.5	2.43
POPE/TMCL 80:20	-	-	6.5	28.8	1.53
LF11-322	-	-	5.6	27.8	1.67
6-MO-LF11-322	-	-	1.9	26.4	1.53
LF11-215	-	-	4.9	27.9	1.74
O-LF11-215	-	-	1.1	27.4	1.73
POPE	-	-	5.1	25.5	1.30
LF11-322			4.8	25.5	1.25
6-MO-LF11-322	-	-	2.2	25.0	1.12
LF11-215	-	-	4.6	25.6	0.99
O-LF11-215	-	-	1.6	25.6	1.89

As outlined earlier PE is a major component of the inner membrane of *E. coli* and thus we also investigated by DSC the effect of the peptides on liposomes composed of POPE/TMCL 80∶20 (wt/wt) (Figure 4, Table 2). In the absence of peptides a broad asymmetric transition was observed at 28.8°C. Addition of the nonacylated peptides at a lipid-to-peptide molar ratio of 25∶1 resulted in a decrease of T_m_ by about one degree accompanied with a slight decrease in enthalpy (Table 2). This indicates a preferential interaction with the higher melting anionic CL leaving a fraction of the membrane enriched in POPE and therefore shifting the remaining phase transition closer to the lower melting component POPE. Upon cooling a splitting of the phase transition was observed (data not shown), supporting the conclusion of formation of POPE enriched and peptide-CL enriched domains being in agreement with earlier observations on similar systems [*14*]; [*15*]. In the presence of the N-acylated peptides the phase transition temperature was further decreased being more prominent for 6-MO-LF11-322 (Table 2). Most interestingly, the enthalpy was dramatically reduced for both N-acylated peptides (Figure 4, Table 2) indicating that they interact with both lipids resulting in a large lipid fraction (70*–*80%) with strongly perturbed hydrocarbon chain order.

Figure 4 also shows the different effects of LF11-322 and 6-MO-LF11-322 on pure POPE liposomes previously described [*2*] with the addition of LF11-215 and O-LF11-215 (Table2) confirming these results. In brief, the nonacylated peptides LF11-215 and LF11-322 exhibited only a marginal effect on the main transition of pure POPE as reflected by a minor decrease of ΔH_m_ by less than 10%. However, both N-acylated peptides 6-MO-LF11-322 and O-LF11-215 again markedly reduced ΔH_m_ by ∼60% (6-MO-LF11-322) and ∼70% (O-LF11-215) respectively. Interestingly, in contrast to the different effects on the main transition of PE and the different effects on cell division, both, nonacylated and N-acylated, peptides abolished the lamellar (fluid L_α_ phase) to inverse hexagonal (H_II_) phase transition (Figure 4). Thus the influence of the N-acylated peptides on the chain melting transition of PE seems to be the main difference that can be observed between the two peptide types.

### Structure of N-acylated peptide O-LF11-215 in SDS micelles

**Figure 5:**
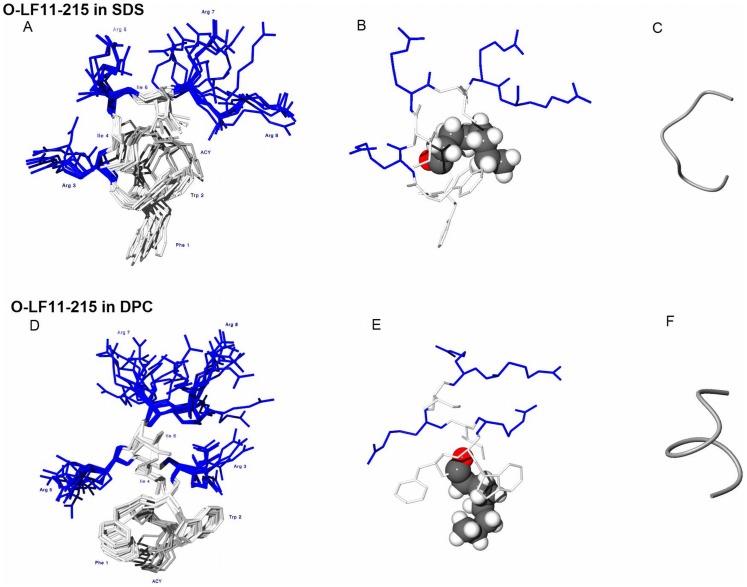
NMR-structures of micelles in presence of N-acylated peptide. NMR structures of O-LF11-215 in complex with SDS (A,B,C) and DPC (D,E,F) micelles. Ensembles of structures are shown in A and D, average structures in B and E and backbone folds in C and F for SDS and DPC micelles, respectively.

In order to investigate potential structural differences induced by N-acylation we studied peptide interaction with different types of membrane mimics using high resolution nuclear magnetic resonance (NMR). SDS was used as forming negatively charged micelles to avoid high noise background of measurements appearing in the presence of phospholipids, High dispersion of O-LF11-215 H^N^-H^α^signals in NOESY and TOCSY indicated a well-defined structure in complex with SDS micelles (Figure 5A-C). Octanoyl group signals were observed at eight frequencies demonstrating different chemical environment for different segments of the acyl chain. R.m.s.d. for backbone atoms of the residues 2*–*7 was 0.66±0.26 Å. The conformation of O-LF11-215 in complex with SDS micelles was not so distinctively α-helical as in the case of O-LF11-215 in complex with DPC micelles (see Figure 5D*–*F), however some parts were in the α-region of the Ramachandran plot. O-LF11-215 adopts a well-defined hydrophobic core (backbone r.m.s.d. (root mean square deviation) of the hydrophobic-core residues is 0.44 Å). Similar to O-LF11-215 in DPC, the structure is amphiphilic with clear distinction between hydrophobic residues forming a wedge and Arg residues geometry complementary to the geometry of negatively charged sulphate groups on the surface of the SDS micelle.

As recently reported by Zorko et al. [*45*] the nonacylated peptide LF11-322 adopts a well-defined structure comprising a short helical segment at different positions in the presence of charged SDS.

O-LF11-215 in DPC micelles

To mimic neutral environment DPC was used in NMR. High dispersion of O-LF11-215 H^N^-H^α^ signals indicated a defined structure also in complex with DPC micelles (Figure 5D-F). Signals for protons from the octanoyl group were observed at 7.3, 7.5, 4.0 and 3.0 ppm. Dispersion of the signals indicates the absence of averaging and a restricted mobility of the C8 chain due to anchoring into the hydrophobic core of the micelle. A number of NOE (nuclear Overhauser effect) signals were observed between octanoyl group protons and Phe^1^, Trp^2^ and Ile^4^. These signals show that the octanoyl group is not extended but is positioned in the “sandwich” between the aromatic rings of Phe^1^ and Trp^2^ residues.

86 inter-residual NOEs were observed among hydrophobic residues including those of octanoyl/Phe^1^, octanoyl/Trp^2^, octanoyl/Ile^4^, Phe^1^/Trp^2^, Phe^1^/Ile^4^, Phe^1^/Ile^6^, Trp^2^/Ile^4^, Trp^2^/Ile^6^ and Ile^4^/Ile^6^. 64 of these NOE connectivities were among the non-sequential residues. The results show that O-LF11-215 has a well-defined hydrophobic core consisting of the octanoyl chain, Phe^1^, Trp^2^, Ile^4^ and Ile^6^. The core is wedge-shaped (Figure 5D*–*F). R.m.s.d. for non-terminal residues 2*–*7 was 0.32 Å, the r.m.s.d. of the hydrophobic residues 0.22 Å. The side chains of basic residues Arg^3^, Arg^5^, Arg^7^ and Arg^8^ point away from the hydrophobic core of the peptide. O-LF11-215 in DPC micelles has an α-helical structure between residues Trp^2^ to Ile^6^. The fact that 5 of 8 residues (62.5%) are in an α-helical structure is in agreement with the secondary structure prediction (60*–*72% calculated using different algorithms such as neural networks, singular value decomposition, regression and self-consistent method, CD data not shown [*46*]–[*50*]). The peptide structure in complex with DPC is amphiphilic consisting of a hydrophobic wedge and Arg basic residues, which form a cluster of positive charge.

As in SDS the nonacylated LF11-322 adopts a well-defined structure with a short helical segment in DPC. However the short α-helical turn is shifted to the N-terminus and less nonpolar surface is exposed than in the presence of SDS [*45*].

### Penetration depth - NMR measurements of the peptide orientation using paramagnetic probes

**Figure 6:**
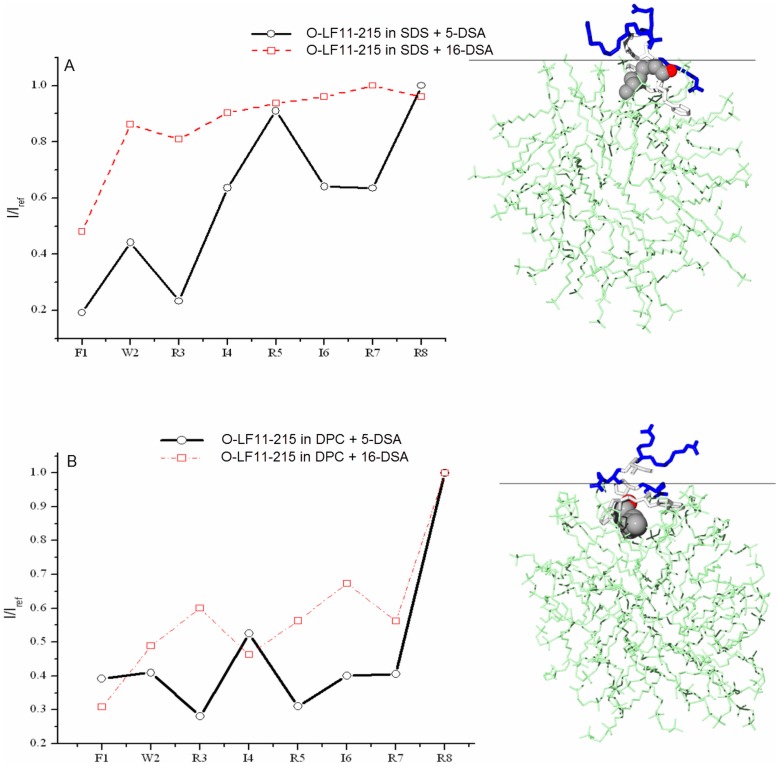
Penetration depth - NMR measurements of the peptide orientation using paramagnetic probes. Normalized H^N^-H^α^ ratios of O-LF11-215 in complex with SDS and DPC micelles (A and B respectively). The figures on the right show the complex of the peptide with the micelle based on the experimental NMR data. Coordinates of the SDS micelle were taken from [*61*]; [*62*]. Tangential line at the surface of the micelle corresponds to the surface of a membrane bilayer.

5-and 16- doxyl stearic acids (5-DSA, 16-DSA) were used as paramagnetic probes to measure the peptide orientation in the complex with SDS and DPC micelles (Figure 6). 5-DSA and 16-DSA caused significant signal broadening (causing signal intensity decrease) of the H^N^-H^α^signals of O-LF11-215 in SDS complex in the N-terminal part of the peptide. The most significant signal decrease was observed for Phe^1^ residue (∼80% signal decrease with respect to the reference spectrum). Deep burial of this residue into SDS micelle interior was confirmed using 16-DSA where the Phe^1^ H^N^-H^α^ signal decreased by 52%. H^N^-H^α^ signal decrease was also observed for Trp^2^ and Arg^3^ residues as well as for Ile^4^ and Ile^6^ residues. This effect however decreased proportionally to the distance from the N-terminus of the peptide is. Arg^3^ residue had much more pronounced paramagnetic relaxation than other Arg residues. This can be explained by interaction between Arg^3^ and sulphate groups at the surface of SDS micelles. This type of interaction was not pronounced for the Arg^5^ and Arg^8^ residues (Figure 6 A).

Significantly more pronounced effect of paramagnetic relaxation enhancement was observed for O-LF11-215 in complex with DPC (Figure 6B) than in complex with SDS micelles. 1/1 molar ratio DSA/SDS micelle was needed to observe the relaxation effect whereas almost all signals disappeared at the same ratio for the DPC micelle. In fact a ratio DSA/DPC of 1/3 was enough to observe significant H^N^-H^α^ signal decrease. The signal intensity decrease due to paramagnetic relaxation was pronounced for the N-terminal part of O-LF11-215 in the presence of DSA, as clearly shown in Figure 6B for the H^N^-H^α^ signal intensity after addition of 16-DSA. The effect was pronounced for Phe^1^, Trp^2^ and Arg^3^. Significant signal ratio decrease was also observed for Ile^4^ suggesting that this residue is buried more than the other residues. Arg^3^, Arg^5^ and Arg^7^ had comparable signal intensity ratios whereas Arg^8^ ratio was 1 suggesting that this residue does not participate in the contact with polar DPC heads and is oriented in solution.

## Discussion

In a previous study we reported mechanistic differences by which N-acylated and nonacylated lactoferricin derivatives kill *E. coli* in correlation with different effects on bacterial lipid model systems [*2*]. N-acylated peptides showed increased membrane perturbation of all bacterial model systems including total bacterial lipid extracts of *E. coli*. In more detailed electron microscopy studies of *E. coli* treated with lactoferricin derived peptides we now observed the formation of membrane blebs resembling those of *E.coli* under stress [*51*] or antibiotic treatment [*23*]. It is known that membrane blebs are released constitutively from many growing Gram-negative bacteria [*52*]–[*55*] and this release is increased under stress [*56*]; [*57*], likely a mechanism that bacteria use to protect themselves, thereby decreasing the amount of a threatening drug. Such mechanism may also be used by the bacteria in these studies as a result of peptide perturbation of cell membranes.

As previously reported hLFcin derivatives cause severe membrane effects that lead to bacterial death before visible membrane lysis [*2*]. In the current study lactoferricin derived lipopeptides, 6-MO-LF11-322 and O-LF11-215, appeared to also have an inhibiting effect on cell division, as deduced from oversized cells of 3*–*5 µm (normal *E. coli* cells exhibit lengths of approx. 2µm (http://redpoll.pharmacy.ualberta.ca/CCDB/cgi-bin/STAT_NEW.cgi)) (Figure 1C, Figure 2 and 3). Peptide induced direct effects on the outer membrane lipids and/or secondary effects on the function of proteins, which show altered activity in an altered lipid environment [*5*], could be triggering such defects in cell division. Similarly, antibiotic treatment reveals inhibition of septum formation and conversion of the cells into filamentous forms, and blebs are formed from the outer membrane, which in some cases leads to leakage of cellular contents into the blebs and further cell lyses [*23*]. A similar morphological change was also shown with LFcin B, where *E.coli* cells became filamentous and elongated after an hour exposure at the peptide MIC [*22*].

During cell division short-lived non-bilayer structures with specific lipid composition appear to be needed to support fusion and fission of lipid bilayers and the activity of proteins involved. Lipids prone to form such phases are cone shaped lipids like PEs. A specific role of PE for growth or viability of *E. coli* cells was already reported [*31*]; [32]; [*58*]. The PE lacking *pss-93* null mutant requires Mg^2+^ or Ca^2+^ for viability [*31*] and *psd* temperature-sensitive mutant with a decreased level of PE and high level of PS [*58*] form long filamentous cells thus demonstrating defect in cell division [*27*]; [*58*].

Inhibition of the transition of PE from lamellar to non-lamellar phases observed in this and previous studies [*2*] might provide evidence that the peptides indeed induce defects in cell division via an effect on the lipid PE. However it does not explain why both peptide types, nonacylated and N-acylated, display this effect on the PE model, but only the N-acylated peptides display the cell division defect. A disordering effect on the hydrocarbon chain packing of PE is however only induced by the N-acylated peptides, which might play a role in inhibiting cell division processes, directly or by hindering the correct formation of CL domains, which was also observed in the present study (Figure 2 and 3) or by deregulating the activity of membrane proteins. Results from Mileykovskaya and coworkers further indicate that either PE itself or wild-type phospholipid composition is required for the formation of the proper FtsZ ring structure, that drives cytokinesis, not to prevent or delay its constriction [*27*].

CL has also been reported to play a role in the dynamic organization of bacterial membranes, e.g., by formation of domains that appear to participate in the regulation of protein activity involved in functions like cell division, where an involvement of CL in the septa formation and constriction in *E. coli* cells was directly demonstrated [*28*]. Also hypothetical models for participation of anionic phospholipids have been implicated with the formation of septal domains in *E. coli* [*29*]; [*59*] and, at cell poles. Enrichment of CL was confirmed later by MS lipid analysis of minicells, which are derived from the cell poles [*60*]. Indeed, as seen by NAO staining of *E. coli* cells incubated in presence of N-acylated peptides, mainly elongated cells were observed in combination with CL-domains being distributed on the whole cell surface, instead of being located at septum and poles, as shown for wild type cells and in the presence of nonacylated peptides (Figure 2 and 3). In our model studies CL organization was influenced by both peptide types, although differently. The effect on the main transition, splitting into two domains in the presence of biologically active nonacylated peptides and strong broadening in the presence of N-acylated peptides, resembled their effect on another negatively charged phospholipid, dipalmitoylphosphatidylglycerol, reported recently [*2*]. These findings support the model [*2*] that the N-acylated peptides induce numerous small membrane domains, creating more defects at domain borders and therefore going in hand with a more severe membrane perturbation as proposed for these peptides when compared to their nonacylated parents.

Our results obtained from liposomes composed of mixtures of PE and CL mimicking the composition of anionic and neutral lipids of the *E. coli* inner membrane demonstrate that the N-acylated peptides strongly interact with the anionic as well as the neutral lipids. In contrast to previous reports on several antimicrobial peptides such as gramicidin S, magainin, PGLa and others [*14*]; [*15*] the N-acylated peptides do not only act by clustering of anionic lipids, as can be seen by the reduction of the T_m_ of the PE/CL mixture, but also influence dramatically the thermotropic phase behavior of PE, revealed by the strong decrease of the enthalpy of the main transition. The strong interaction of N-acylated peptides with CL and PE might hinder the formation of CL-domains at septum and poles as observed by NAO staining (Figure 2 and 3). It has been discussed previously that cardiolipin clustering by antimicrobial peptides out of mixtures of CL and PE, which in our studies seems to be strongest by N-acylated peptides (higher T_m_ shift), might be partially responsible for an arrest of cell growth or cell death of bacteria [*13*]; [*14*]. Second, the fact that PE which is proposed to play an important role in the cell division cycle that cannot be completely compensated by CL [*32*] lets suggest a further cause of defects in cell division in our experiments by the finding that the N-acylated peptides additionally severely perturb PE membranes.

NMR data support this different type of interactions between lipids and nonacylated vs. N-acylated peptides, respectively. As shown by Zorko et al. [*45*] LF11-322 adopts a well-defined structure comprising a short helical segment at different positions in the presence of charged SDS-micelles and zwitterionic (non-charged) DPC-micelles. In SDS the backbone conformation of the peptide forms a short α-helical turn between residues Trp^3^ and Arg^6^ with more nonpolar surface exposed than in the presence of DPC where the short α-helical turn is shifted to the N-terminus and is positioned between residues Phe^2^ and Ile^5^. The α-helical axis of the lactoferricin derived peptides in a membrane bilayer was predicted by Zorko et al. [*45*] to lie perpendicular to the membrane plane, which is in contrast to longer α-helical peptides lying parallel to the bilayer plane. The three hydrophobic residues at the N-terminus of LF11-322 are reported to form a cluster, which is more compact in the zwitterionic environment but has a larger lateral dimension in the presence of anionic micelles. Such a cluster seems to contribute to the discrimination between bacterial and eukaryotic membranes [*45*]. The cationic residues Arg^4^ and Arg^6^ are proposed to contribute to embedding the peptide deeper into the SDS micelle. In contrast to the nonacylated peptides in this study, the N-acylated peptide O-LF11-215 exhibits a change of structure in the presence of both SDS and DPC, with a larger proportion of α-helix in the presence of DPC (CD data, not shown). Interestingly, more of the peptide amino acids penetrate into neutral micelles than in charged ones. This fits with the model in which both charged and neutral lipids on the bacterial membrane are affected by N-acylated peptides, likely leading to enhanced membrane defects and effects on cell division. A correlation of membrane penetration depth and biological activity was also previously reported for derivatives of bovine LFcin [*20*]. Based on solution NMR of an 11-mer of bovine LFcin bound to SDS, it has been suggested that the cyclic form of the 11-mer exhibits higher antimicrobial activity than its linear counterpart due to the fact that the cyclic peptide Trp residues appeared embedded deeper into the membrane. While both peptides adopted an amphipathic structure without any regular α-helical or β-sheet conformation, the 3D-structures revealed a clearer partitioning of the cationic and hydrophobic faces for the cyclic peptide [*20*].

Overall it can be proposed that N-acylated peptides besides killing bacteria via strong membrane perturbation interact with bacterial lipids in a way that causes direct or indirect defects in cell division. Mainly the effect on both CL and PE seems to be responsible for the inability of CL domains to form for proper proliferation. The study shall help clarifying the multiple mechanisms by which antimicrobial peptides can act on bacterial membranes to improve peptide activity and specificity.
