# Correction: N-acylated Peptides Derived from Human Lactoferricin Perturb Organization of Cardiolipin and Phosphatidylethanolamine in Cell Membranes and Induce Defects in *Escherichia coli* Cell Division

**DOI:** 10.1371/journal.pone.0099324

**Published:** 2014-05-29

**Authors:** The PLOS ONE Staff

There are errors in the funding statement. The correct funding statement is as follows: This work was supported by EC-specific RTD Program Grant QLK2-CT-2002-01001. RJ, BJ and MZ were supported by the program and projects from the Slovenian Research Agency and in part by the European structural funds to the Centre of Excellence EN-FIST. This work was also supported by NIH grant GM020478 awarded to WD. The funders had no role in study design, data collection and analysis, decision to publish and preparation of the manuscript.
